# Quality of pre-service midwifery education in public and private midwifery schools in Afghanistan: a cross sectional survey

**DOI:** 10.1186/s12909-021-03056-1

**Published:** 2022-01-16

**Authors:** Partamin Manalai, Sheena Currie, Massoma Jafari, Nasratullah Ansari, Hannah Tappis, Faridullah Atiqzai, Young Mi Kim, Jos van Roosmalen, Jelle Stekelenburg

**Affiliations:** 1 Athena Institute, Vrije Universiteit, Amsterdam, The Netherlands; 2 Jhpiego, 1615 Thames Street, Baltimore, MD 21231 USA; 3 Afghan Midwives Association, HNO5, Baharistan, 2th District, Kabul, Afghanistan; 4 UNDP, Merkez, Abide-i-Hurriyat Cd 142, 34381 Istanbul, Turkey; 5 Department of Health Sciences, Global Health, University Medical Centre Groningen/University of Groningen, PO Box 196, 9700 AD Groningen, The Netherlands; 6 Department of Obstetrics and Gynaecology, Leeuwarden Medical Centre, Henri Dunantweg 2, 8934 AD Leeuwarden, The Netherlands

**Keywords:** Midwifery, Pre-service education, Quality, Afghanistan

## Abstract

**Background:**

Midwives are the key skilled birth attendants in Afghanistan. Rapid assessment of public and private midwifery education schools was conducted in 2017 to examine compliance with national educational standards. The aim was to assess midwifery education to inform Afghanistan Nurses and Midwives Council and other stakeholders on priorities for improving quality of midwifery education.

**Methods:**

A cross-sectional assessment of midwifery schools was conducted from September 12–December 17, 2017. The Midwifery Education Rapid Assessment Tool was used to assess 29 midwifery programs related to infrastructure, management, teachers, preceptors, clinical practice sites, curriculum and students. A purposive sample of six Institute of Health Sciences schools, seven Community Midwifery Education schools and 16 private midwifery schools was used. Participants were midwifery school staff, students and clinical preceptors.

**Results:**

Libraries were available in 28/29 (97%) schools, active skills labs in 20/29 (69%), childbirth simulators in 17/29 (59%) and newborn resuscitation models in 28/29 (97%). School managers were midwives in 21/29 (72%) schools. Median numbers of students per teacher and students per preceptor were 8 (range 2–50) and 6 (range 2–20). There were insufficient numbers of teachers practicing midwifery (132/163; 81%), trained in teaching skills (113/163; 69%) and trained in emergency obstetric and newborn care (88/163; 54%). There was an average of 13 students at clinical sites in each shift. Students managed an average of 15 births independently during their training, while 40 births are required. Twenty-four percent (7/29) of schools used the national 2015 curriculum alone or combined with an older one. Ninety-one percent (633/697) of students reported access to clinical sites and skills labs. Students mentioned, however, insufficient clinical practice due to low case-loads in clinical sites, lack of education materials, transport facilities and disrespect from school teachers, preceptors and clinical site providers as challenges.

**Conclusions:**

Positive findings included availability of required infrastructure, amenities, approved curricula in 7 of the 29 midwifery schools, appropriate clinical sites and students’ commitment to work as midwives upon graduation. Gaps identified were use of different often outdated curricula, inadequate clinical practice, underqualified teachers and preceptors and failure to graduate all students with sufficient skills such as independently having supported 40 births.

**Supplementary Information:**

The online version contains supplementary material available at 10.1186/s12909-021-03056-1.

## Background

Afghanistan has come a long way in reducing the maternal mortality ratio (MMR) from 1600 in 2002 to 638 per 100,000 live births in 2019 [1, 2]. Female clinicians (midwives, female doctors and obstetrician &gynecologists) were present in only 138 (18%) of the 783 public health facilities However, Package of Health Services (BPHS) with 1075 health facilities in 2004 and 1829 in 2011 [3]. MoPH invested in educating competent midwives with the essential range of skills and recruited them to increase the numbers of skilled birth attendants (SBA) since early 2003 [4]. This included strengthening of 2 years’ diploma midwifery education programs through the existing Institute of Health Sciences (IHS) and establishing community midwifery education (CME) programs, all compliant with the International Confederation of Midwives (ICM)’ recommendations on core competencies in midwifery [5]. The schools were funded by MoPH, and several bilateral and multilateral international non-governmental organizations (NGOs). The number of midwives working in Afghanistan was only 467 in 2002 and this number increased to 7244 in 2019 [6, 7]. Private schools graduated 25,177 midwives during the period from 2009 to 2019 [8]. There are, however, no reliable data about the employment status of the midwives graduated from private schools. With a crude birth rate of 35 per 1000 persons or 1.2 million children born per year in Afghanistan (total population 32.2 million) and at a rate of 4.45 SBA per 1000 as recommended by the World Health Organization (WHO), the number of midwives may look sufficient nowadays [9–11]. Midwives’ availability, however, was 16.7% in public facilities in Southeastern provinces as compared to 63.6% in Northeastern provinces indicating inequity in their distribution [12]. Although, broader health system, socio-cultural and security issues are important, high quality midwifery education remains nevertheless crucial to addressing people’s access to midwifery care in Afghanistan [13].

MoPH developed a national accreditation policy based on educational standards and established the Afghanistan Midwifery and Nursing Education Accreditation Board (AMNEAB) in 2005 [14]. An evaluation of public midwifery schools in 2008 identified areas of strengthening midwifery programs as ICM and MoPH recommended and followed by establishment of a national 2 years’ curriculum for IHS and CME programs [15]. Competency building, effective preceptorship, simulated and clinical practice, however, remained poorly documented [16].

A systematic evaluation of midwifery education has not been carried out to examine its various dimensions. Midwifery education became a priority for assessment with the support of MoPH, donors such as the United States Agency for International Development (USAID), AMA and other partner organizations as part of the USAID-funded project HEMAYAT. This paper is the result of a rapid assessment of public and private midwifery schools in the end of 2017, conducted in collaboration with Jhpiego, AMA, MoPH, in a time that efforts were at its peak to establish the Afghanistan Nurses and Midwives Council (ANMC) with the mandate to assure high quality preservice education for midwives [17]. The objective of the study was to examine public and private school educational standards for infrastructure and management, numbers and competencies of teachers and preceptors, clinical practice sites, curriculum and numbers of students per class. Findings will help stakeholders to inform future policies and practices towards improved quality of midwifery education in Afghanistan.

## Methods

### Research design

We conducted a cross-sectional assessment of public and private midwifery schools from September to December 2017. The Midwifery Education Rapid Assessment Tool developed by Jhpiego was adapted for use in Afghanistan and included interview tools for school managers, teachers and preceptors, a self-administered questionnaire for midwifery students and a clinical practice site observation form. Tools were pre-tested in two private schools in Kabul in August 2017 [18]. This tool, based on ICM’s educational standards, was designed to assess five areas of pre-service midwifery education: infrastructure and management, teachers and preceptors, clinical practice sites, curriculum and students. Review of school records and documents, interviews and observation of school and clinical practice facilities were the research methods used . The study was approved by both Institutional Review Boards of the Afghan Public Health Institute and the Johns Hopkins Bloomberg School of Public Health.

### Setting

**Fig. 1:**
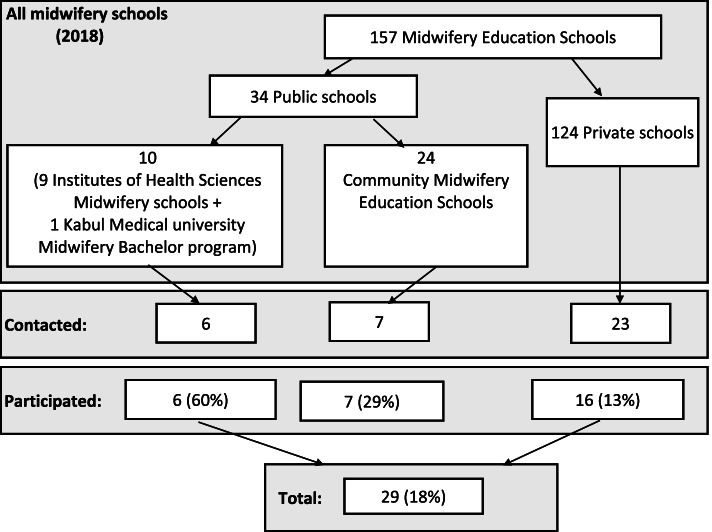
Midwifery schools in the rapid assessment

Afghanistan had eight IHS, one direct-entry bachelor’s degree midwifery program, implemented by Kabul Medical University (KMU) (also treated as IHS in this assessment), 24 CME and 124 private midwifery schools at the time of assessment (Fig. 1). IHS midwifery schools accept 12th grade school students through a yearly entry course examination and provide a 2 years’ diploma curriculum for midwives to serve primarily in large clinical and hospital settings after graduation. The KMU program also accepts students through concourse and provides a 4 years bachelor’s curriculum. CME schools accept candidates from communities, selected through a community dialogue from the eligible school graduate girls and women in special CME schools located in provinces. Graduates of CMEs are bound by a commitment and community guarantee to work at least 2 years in primary health care clinics, designated at the start of the training. However, CME graduates are also able to work in larger clinics and hospitals. Private schools in this assessment implement 2 years’ midwifery diploma programs.

Schools were purposively selected from five large population provinces, namely Kabul, Balkh, Herat, Nangarhar and Kandahar to reflect school type and funding sources. The five IHS from these provinces with KMU were included (total 6 IHS). One CME from Kabul, Herat and Kandahar were included, but Balkh and Nangarhar provinces did not have CME-schools, therefore, the nearest CME programs (i.e. Baghlan instead of Balkh and Laghman instead of Nangarhar) were selected. As all schools were supported either by the government or by USAID, two CME schools funded by other donors, both in Faryab province, were also included (total 7). Due to accessibility and security constraints in the five focus provinces, only 23 out of 124 private schools could be contacted, out of which 16 agreed to participate in the study. Sampling was nonrandom, and no statistical testing was intended.

#### Data collection

Data collectors were ten Jhpiego Provincial Midwifery Officers and four members of the Afghan Midwives Association (AMA), all female midwives. They were trained to use data collection tools, research methodology and ethics for 5 days. Teams of 2–3 data collectors visited each school for 2 days from September–December 2017. They reviewed register books and school records, visited a maximum of three on-going classes, visited clinical practice sites and conducted interviews with target participants. The assessment team monitored the data collectors in the field and data were checked for consistency. Jhpiego’s Monitoring Evaluation and Research Manager conducted interviews with school managers, teachers and clinical preceptors. Data collectors asked students to fill in self-administered questionnaires. They observed school premises and school records, student logbooks and clinical registers to confirm data on infrastructure, management, curriculum and equipment.

#### Analysis

Two members of the team entered and cleaned quantitative data in a Microsoft Excel database, transferred to STATA IC 15 for analysis. The assessment team lead completed quantitative analysis including calculation of counts, percentages, means and standard errors, medians and ranges for different school and health facility types. Preliminary findings of the survey were presented by the research team to school representatives and key stakeholders led by the MoPH in a validation workshop in March 2018.

## Results

Seven CME, six IHS (including the KMU bachelor program) and 16 private schools were assessed. Median number of years of activity was 7.6 (range 1.7–12) for CME, 39.5 (range 3.8–45.9) for IHS and 5.8 for private schools (range 1.6–10.8). The KMU program had been in operation for 4 years, but had not graduated any student at the time of assessment. Median number of students ever graduated from the selected schools was 67 for CME (range 25–106) and 406 for IHS (range 251–692); numbers of students graduated from private schools were unavailable.

The assessment identified gaps in infrastructure, availability and preparedness of the teachers and preceptors, students’ access to clinical sites and practical work in relation with caseloads and inconsistencies in curricula in the different schools.

### Infrastructure, equipment and management

More than 90% (26–28/29) of the schools had rooms for classes, desks for every student, classrooms with good ventilation and lights and unobstructed views for students. All schools accommodated no more than 30 students in one room. Fourteen out of 29 schools (48%) met all six classroom criteria (Table 1). While 28 (97%) schools had a library, only four (14%) had all nine recommended books (supplementary file 1). All schools reported having skills labs, which were actually open for students at the time of assessment in 20 (69%) and 22 (76%) had full-time skills lab managers. Childbirth simulators were available in 17 (59%) schools and all except one private school had newborn resuscitation models. Midwives served as managers in 21 (72%) schools and 22 (76%) managers had experience in midwifery, including one doctor who had been a midwife in the past (Table 1).

**Table 1:** Infrastructure and available equipment during assessment visit, by school type

	CME (***n*** = 7)	IHS (***n*** = 6)	Private (***n*** = 16)	Total (***n*** = 29)
**Classroom facilities**				
Desks for every student	7	6	15	28 (97%)
Elbow room for students	6	5	14	25 (86%)
Classrooms with sufficient light and ventilation	7	6	14	27 (93%)
Unobstructed view of subject matter in classrooms	7	6	13	26 (90%)
Specific teaching rooms	7	6	14	27 (93%)
Accommodation for 30 students per class	7	3	10	20 (69%)
Schools meeting all six criteria	6	2	6	14 (48%)
**Library**				
Library exists	7	6	15	28 (97%)
Library is open after official hours and during weekends	6	1	3	10 (34%)
All nine recommended textbooks available (listed in Supplementary file 1)	1	2	1	4 (14%)
**Skills lab**				
Skills lab open for individual/group practice	6	6	8	20 (69%)
Schools with full-time skills lab manager	6	6	10	22 (76%)
**Computer lab**				
Schools with computer lab	6	5	11	22 (76%)
Schools with one computer for every 10 students	5	5	8	18 (62%)
Schools with Internet connection	2	3	6	11 (38%)
**Equipment**				
Childbirth simulator (electronic manikin childbirth simulator, MamaNatalie, Noelle)	5	5	7	17 (59%)
Bony pelvis model	7	6	15	28 (97%)
Breast model	5	5	11	21 (72%)
Cervical dilatation model	5	6	4	15 (52%)
Fetal skull model	6	4	4	14 (48%)
Intrauterine contraceptive device insertion model	6	6	14	26 (90%)
Newborn resuscitation models, including NeoNatalie	7	6	15	28 (97%)
Perineum cutting and suturing simulators	5	4	6	15 (52%)
Pelvic model	7	6	13	26 (90%)
Implant insertion and removal kit	0	1	1	2 (7%)
Vaginal speculum	7	6	16	29 (100%)
Delivery kits	7	6	14	27 (93%)
Sterilizer	5	4	7	16 (55%)
Video or DVD player (may be located in computer lab) and associated teaching videos	5	4	11	20 (69%)
Thermometer in working order	7	6	15	28 (97%)
Antiseptic solutions	7	5	15	27 (93%)
Running water and soap and/or hand sanitizer	7	5	14	26 (90%)
**Teachers’ office(s)**				
Electricity	7	6	16	29 (100%)
Running water	6	5	15	26 (90%)
Necessary supplies	7	5	12	24 (83%)
Related textbooks	7	4	12	23 (79%)
Desks for teachers	4	2	4	10 (34%)

### Teachers and clinical preceptors

Observational visits to one, two or three ongoing classrooms were conducted when available. In these visits, median number of students per teacher was 8.3 (range 1.8–50) and students per preceptor was 6 (range 1.5–20). All IHS schools assigned specific personnel as clinical preceptors, while 6/7 (86%) CME schools and 11/16 (69%) private schools did so (Table 2).

**Table 2:** Student-to-teacher and student-to-preceptor ratios

	CME (n = 7)	IHS (n = 6)	Private (n = 16)	Total (n = 29)
**Number of classrooms observed**				
One	3	0	1	4
Two	2	3	1	6
Three	2	3	12^a^	17
**Theory-based classes**				
Number of students	252	630	1429	2311
Number of teachers	38	51	188	277
Median students-to-teacher ratio (range)	7.0 (4.0–20.0)	15.5 (6.0–43.3)	10.9 (1.8–50.0)	8.3 (1.8–50.0)
Number (%) of schools meeting criterion of one teacher per 30 students	7 (100%)	4 (67%)	13 (81%)	24 (83%)
**Practical studies**				
Number of schools with preceptors at their clinical sites	6 (86%)	6 (100%)	11 (69%)	23 (79%)
Number of students at school clinical sites	134	112	77	323
Number of preceptors at clinical sites	21	13	22	56
Median student-to-preceptor ratio (range)	5.0 (4.0–7.0)	7.3 (2.0–20.0)	4.0 (1.5–10.0)	6.0 (1.5–20.0)

^a^ Two schools were excluded reporting unrealistically large student numbers considered error

**Fig. 2:**
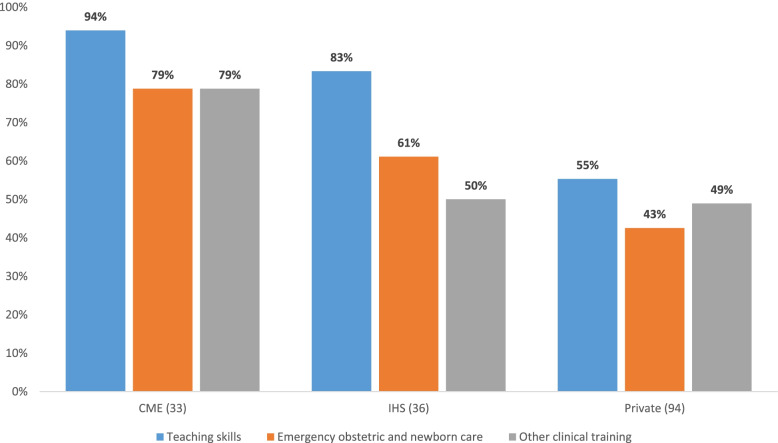
Percentage of teachers who have received the selected trainings by school type

The assessment team conducted interviews with 163 teachers. From this group, 132 (81%) had practiced midwifery and 150 (92%) had previous teaching experience. In total, 95 (58%) teachers had been practicing midwifery for at least 2 years. Of 163 teachers, 113 (69%) had been trained in teaching skills and 88 (54%) in emergency obstetric and newborn care. Student-to-teacher and student-to-preceptor ratios per school type are shown in Fig. 2.

A subgroup of 58 out of 163 (36%) teachers were willing to discuss their teaching practices with none of them reporting use of role-play as a teaching method. In addition, 51 clinical preceptors were assigned to the selected schools with 25 (49%) having no other parallel assignments in clinics and 41 (80%) preceptors received coaching from school teachers (Table 3).

**Table 3:** Preparedness and competency of teachers

	CME (n = 7)	IHS (n = 6)	Private (n = 16)	Total (n = 29)
**Number of teachers interviewed**	**33**	**36**	**94**	**163**
Range of teachers per school	3–6	3–10	2–10	2–10
**Background information of contacted teachers**				
Teachers ever practiced midwifery	29 (88%)	27 (75%)	76 (81%)	132 (81%)
Mean number of years of clinical practice	5.0	4.9	4.0	4.4
Teachers with at least 2 years of clinical practice	21 (64%)	21 (58%)	53 (56%)	95 (58%)
Teachers with previous teaching experience	28 (85%)	32 (89%)	90 (96%)	150 (92%)
Mean number of years teaching	3	6	4	4
Percentage of teachers with at least 2 years of teaching experience	19 (58%)	26 (72%)	70 (74%)	115 (71%)
Teachers with previous management experience	14 (42%)	9 (25%)	15 (16%)	38 (23%)
Mean number of years in management	1.8	1.9	0.7	1.2
**Training received**				
Teaching skills	31 (94%)	30 (83%)	52 (55%)	113 (69%)
Emergency obstetric and newborn care	26 (79%)	22 (61%)	40 (43%)	88 (54%)
Other clinical training*	26 (79%)	18 (50%)	46 (49%)	90 (55%)
**Number of teachers who responded to questions about teaching practices**	**14 (42%)**	**12 (33%)**	**32 (34%)**	**58 (36%)**
**Teaching methods reported**				
**Knowledge acquisition**				
Lecture/presentation with group participation	13 (93%)	11 (92%)	32 (100%)	56 (97%)
Project-based learning	12 (86%)	10 (83%)	23 (72%)	45 (78%)
Seminar /discussion	12 (86%)	9 (75%)	15 (47%)	36 (62%)
Group work	11 (79%)	7 (58%)	14 (44%)	32 (55%)
Role-play	0 (0%)	0 (0%)	0 (0%)	0 (0%)
**Clinical decision-making**				
Case studies	10 (71%)	7 (58%)	11 (34%)	28 (48%)
Problem-based learning	8 (57%)	8 (67%)	20 (63%)	36 (62%)
**Skills acquisition**				
Skills demonstration	9 (64%)	9 (75%)	16 (50%)	34 (59%)
Skills practice	4 (29%)	3 (25%)	5 (16%)	12 (21%)
Clinical simulated practice	3 (21%)	4 (33%)	2 (6%)	9 (16%)
**Number of clinical preceptors contacted**	**14**	**12**	**32**	**58**
**Number of clinical preceptors consented to participate**	**14 (100%)**	**11 (92%)**	**26 (81%)**	**51 (88%)**
Serve as preceptor with no other assignments	6 (43%)	4 (36%)	15 (58%)	25 (49%)
Practice predetermined number of cases together with students	6 (43%)	2 (18%)	8 (31%)	16 (31%)
Assigned to work on more than two cases of childbirth with each student (exempted from other tasks)	8 (57%)	6 (55%)	15 (58%)	29 (57%)
Assigned to work one on one with each student	10 (71%)	7 (64%)	20 (77%)	37 (73%)
Works with maximum two students per shift	8 (57%)	8 (73%)	20 (77%)	36 (71%)
Relief of workload while working with students	9 (64%)	6 (55%)	19 (73%)	34 (67%)
Supported by academic faculty during work with students	12 (86%)	9 (82%)	20 (77%)	41 (80%)

* Including sexually transmitted infections, family planning, mental health, postpartum hemorrhage, eclampsia and pre-eclampsia, interpersonal communication, Essential Care for Sick Babies, postpartum intrauterine contraceptive device, Helping Babies Breathe, Helping Babies Survive and epidemiology,

### Clinical sites

Schools had agreements with between one and 15 health facilities (median 3) serving as clinical practice sites for their students. The standard of assisting 40 uncomplicated births for graduation was mentioned in 16/29 (55%) schools. Median number of births recorded as independently practiced, was 40 births (range 0–70) per student (Table 4).

**Table 4:** Use of clinical sites by school type

	CME (n = 7)	IHS (n = 6)	Private (n = 16)	Total (n = 29)
Schools with clinical sites for practical studies	7 (100%)	6 (100%)	16 (100%)	29 (100%)
Median number of health facilities used as clinical practice sites (range in bracket)	3 (1–8)	5.5 (1–8)	3 (1–15)	3 1–15)
Median number of births assisted at graduation as reported by the clinical sites. (range in bracket)	41 (40–50)	40 (0–70)	22.5 (0–60)	40 (0–70)
School clinical sites policy requiring students to perform minimum of 40 births independently for graduation	7 (100%)	4 (67%)	5 (31%)	16 (55%)
Schools with clinical sites not located in the same vicinity	5 (71%)	5 (83%)	10 (63%)	20 (69%)
Schools providing transport for students and teachers to commute to clinical sites	6 (86%)	5 (83%)	13 (81%)	24 (83%)

For each school, one facility used as clinical site was selected including one basic health center, five comprehensive health centers, five provincial hospitals, 11 private health facilities, five regional hospitals and two specialized hospitals. These 29 facilities reported 90,297 uncomplicated births in the past 6 months. On average, 13 students were accommodated in 8 h working shifts in these facilities.

In a subset of 20 (69%) clinical sites facilities, data collectors were also allowed to review student logbooks showing that students assisted on average 14.6 births with the highest average of 40 for two SH. In this subgroup, preceptors in 4/20 (20%) health facilities (2 PH and 2 SH) declared that completing assistance of 40 births is enforced for graduation (Table 5).

**Table 5:** Capacity of clinical practices sites, by facility type

Clinical site readiness	BHC (n = 1)	CHC (***n*** = 5)	PH (n = 5)	Private (***n*** = 11)	RH (***n*** = 5)	SH (n = 2)	Total (n = 29)
**Availability of selected inputs**							
Number of midwives and nurses at day shift	1	6	28	49	29	8	121
Number of midwives and nurses at night shift	0	5	16	24	23	6	74
Sphygmomanometer	1	5	5	10	5	2	28 (97%)
Pinard/fetal stethoscope/Doppler (in any combination)	0	5	5	10	5	2	27 (93%)
Gloves	1	5	5	8	5	2	26 (90%)
Uterotonics (oxytocin or alternative)	1	5	5	8	5	2	26 (90%)
IV solution and IV set	1	5	5	8	5	1	25 (86%)
Sterile birth kit	1	4	5	9	5	2	26 (90%)
Decontamination solution	1	4	5	9	5	2	26 (90%)
Newborn resuscitation bag and mask	1	5	5	9	5	2	27 (93%)
Clinical guidelines available	0	4	5	3	2	2	16 (55%)
**Clinical services utilization in past 6 months**							
Uncomplicated births	52	21,526	8013	5250	36,578	18,878	90,297
Assisted vaginal births	2	2698	3007	382	2471	1358	9918
Cesarean section	0	7417	684	533	10,378	5613	24,625
Antenatal care	1096	8369	6577	11,969	22,367	1689	52,067
Postnatal care	225	3581	3670	5938	31,895	619	45,928
Family planning	572	4258	3298	1929	21,543	13,973	45,573
**Educational capacity**							
Median number of students per shift (range)	4	6 (6–7)	12 (6–25)	15 (4–32)	6 (3–12)	31 (18–44)	7 (3–44)
Number of schools that use this facility as clinical site	1	13	16	19	16	36	–
Used by one school	1	1	1	8	1	–	12
Used by two to three schools	–	3	2	1	1	–	7
Used by four to five schools	–	1	2	2	3	–	8
Used by 6–21 schools	–	–	–	–	–	2	2
**Student checkout for competency in uncomplicated birth (for facilities where student logbooks were available for review)**							
Number of facilities where student logbooks were reviewed	1 (100%)	4 (80%)	4 (80%)	8 (73%)	1 (20%)	2 (100%)	20 (69%)
Median number of conducted births student logbook showed till the time of graduation	1	16.5	26.5	0	0	40	14.6
Students conducted 40 births independently for graduation/out of total logbooks observed	0/1	1/4	1/4	0/8	0/1	2/2	4/20 (20%)

### Curriculum

Different editions of midwifery curricula were in use in the schools including IHS 2006 midwifery curriculum, IHS 2010 midwifery curriculum, CME 2010 midwifery curriculum and national 2015 midwifery curriculum. Two (13%) private schools were using their own customized curriculum. Seven (24%) schools were using the national 2015 midwifery curriculum among which one IHS used IHS 2010 and one private school also used IHS 2006 complementary. Five (71%) CMEs used CME 2010 curriculum, while 13 (45%) schools including one CME used IHS 2010 curriculum. One (17%) private school used IHS 2006 and KMU Bachelor program had its own curriculum.

Teaching materials for curriculum implementation were provided by MoPH for 27 schools, while the KMU Bachelor program and one private school developed their own learning materials.

### Students

Self-administered questionnaires were given to 697 students from 28 schools. One CME school declined to participate. Access to clinical sites was reported by 633 (91%) students, access to skills labs by 631 (91%) and access to computer labs by 457 (66%). Barriers cited by students were lack of access to health facilities, low caseload, lack of equipment and supplies, insufficient number of preceptors and being neglected by the teachers and preceptors tending to prioritize other clinical and administrative duties. All 116 CME students said they will work as midwives upon graduation; while 117 of 131 IHS students (89%) and 384 of 450 private school students (85%) stated such intention (Table 6).

**Table 6:** Education experience of students

	CME (n = 6)	IHS (n = 6)	Private (n = 16)	Total (n = 29)
**Respondents**				
Number of students interviewed	116	131	450	697
Mean number of students per school	19	22	28	25
**Education experience**				
Students had access to facilities at clinical site	115 (99%)	111 (85%)	407 (90%)	633 (91%)
*Reasons for students with no access*				
Lack of cases due to low caseload		1	2	3
Lack of equipment and supplies		5	2	7
Neglect or prohibition		1	2	3
Insufficient preceptors		2	0	2
No details	1	11	37	49
Students had access to facilities at skills lab	115 (99%)	116 (89%)	400 (89%)	631 (91%)
*Reasons for students with no access*				
Lack of equipment and supplies	0	1	0	1
Neglect or prohibition	0	1	1	2
No details	1	13	49	63
Students had access to facilities at computer lab	94 (81%)	89 (68%)	274 (61%)	457 (66%)
*Reasons for students with no access*				
Lack of equipment and supplies	8	5	18	31
Neglect or prohibition	0	0	1	1
Male dominated usage	0	0	1	1
No details	14	37	156	207
Students feel safe and secure	112 (97%)	123 (94%)	421 (94%)	656 (94%)
Students feel ready to work as midwives	116 (100%)	117 (89%)	384 (85%)	617 (89%)
Students who reported miscellaneous challenges				
Lack of preceptors	0 (0%)	16 (12%)	5 (1%)	21 (3%)
Lack of clinical work	8 (7%)	25 (19%)	91 (20%)	124 (18%)
Prohibition and disrespect	0 (0%)	10 (8%)	4 (1%)	14 (2%)
Lack of equipment and supplies	2 (2%)	3 (2%)	10 (2%)	15 (2%)
Lack of transport facilities	0 (0%)	19 (15%)	12 (3%)	31 (4%)
Poor quality of clinical work	0 (0%)	0 (0%)	2 (0%)	2 (0%)
Low caseload due to costs	0 (0%)	0 (0%)	3 (1%)	3 (0%)
Clients refuse to cooperate	0 (0%)	0 (0%)	2 (0%)	2 (0%)
**Motivation and concerns**				
Students themselves decided to study midwifery	112 (97%)	123 (94%)	400 (89%)	635 (91%)
Students plan to work as midwives after graduation	111 (96%)	124 (95%)	423 (94%)	658 (94%)
Students have a specific facility in mind to work in	80 (69%)	75 (57%)	242 (54%)	397 (57%)
Students concerned about employment	1 (1%)	23 (18%)	89 (20%)	113 (16%)
Students concerned about career pathway	(0%)	(0%)	5 (1%)	5 (1%)

## Discussion

Our findings indicate that public and private midwifery schools are preparing midwives with mixed results. Some expectations are met by the programs in different areas. Specifically, classrooms, skills labs and clinical practice sites of most schools had the required infrastructure, equipment and supplies. The majority of schools used one of the once-approved versions of the curricula, though some were outdated. Midwives were in leading positions in most schools and most teachers had experience in midwifery or management. Students, especially in CME schools, reported feeling safe and secure in their schools and were determined to work as midwives in the future. This is encouraging in the light of findings of a study in 11 provinces showing employment rates for CME graduates from 28.4% (in Khost province) to 84.3% (in Herat province) [19]. Recruitment into CME programs is aligned with a health workforce approach to encourage retention and commitment to serve their own communities. Several shortcomings, however, were identified by that study including inadequate resources and incompetence of the teachers and preceptors. Midwives may graduate from these schools without meeting certain global or national competency requirements and the ability to perform life-saving interventions with confidence as mandated by ICM [20].

Shortages of learning materials, teachers and preceptors and overburdened clinical sites were identified in several schools, while low caseloads in smaller facilities were also observed. Number of students per teacher varied largely and was as high as 50 in one school. One teacher per 45 students has also been observed in other low-income countries [21]. Shortage of teachers can deprive students of support and interaction and compromises quality education. Some schools with higher numbers of teachers may have many part-time teachers serving in different schools. This may affect the level of attachment to specific cohorts of students and compromises commitment and accountability for competency building of students. Knowing that optimizing teacher-student ratios requires additional investment, ANMC and MoPH should ensure such investments are made. Some schools in Afghanistan tried to employ new graduates to fill these gaps with rather unexperienced teachers [22]. MoPH and ANMC, however, have to verify that schools are established with sufficient numbers of competent teachers and preceptors and advocate to focus on quality and quantity of faculty per standards. Only then is Afghanistan in a better position to meet Sustainable Development Goal 3 to improve maternal and newborn health, as emphasized by Strengthening Quality Midwifery Education for Universal Health Coverage in 2030 [23].

Competencies of teachers and preceptors were questionable with many of them having no training in evidence-based clinical and teaching methodologies, lecturing in a traditional way instead of more interactive student-centered methods. Poor teaching and clinical skills of midwifery faculties and preceptors are commonly found in low- and middle-income countries as was shown in Ethiopia by documented dissatisfaction of students [24]. Regular capacity assessments and continuing education are required to keep teachers up-to-date with standards and evidence-based clinical practices [25]. ANMC should monitor the maximum number of students per teacher and preceptor and require schools to demonstrate their investments in continued education of their faculties [26].

Complacency with achieving ICM competencies will lead to less educated midwives who are not able to provide high-quality care [27]. The 2015 national midwifery curriculum requires students to independently perform 40 births to become competent and competency-based education is the basis of midwifery education in Afghanistan [28]. A review of 73 countries showed that more than 30 births assisted by students occurred in 32 (44%) of them, implying similar constraints globally [29]. ANMC are in a unique position as they established regulatory systems to learn from experiences of other countries in ensuring midwives to be competent at graduation. All necessary elements of high-quality midwifery care must be taught, balancing theory and practice to produce fully competent midwives upon graduation [30].

Midwifery care is cost-effective, affordable and sustainable. It has contributed to improvement of maternal and newborn health [31, 32]. Midwifery reduces maternal and newborn deaths and stillbirths, strengthens economic activity and ripples favorably across macroeconomics, provides women with decent work and results in economic stabilization in society [33]. Specifically, midwifery leads to better health outcomes. Insufficient monitoring of midwifery education is recognized by the global community as a major area of concern [23]. In Afghanistan, midwifery education was not explicitly mentioned among high-priority areas in the 2011 draft national policy on nursing and midwifery [34]. Due to lack of strong positive and direct language in the policy it is difficult to encourage clinical facilities to willingly and enthusiastically accommodate learning opportunities for student-midwives.

Clinical sites, often independent of the schools, do not bear the responsibility of providing sufficient clinical work for students [35]. On the other hand, clinical sites face challenges with simultaneously competing students, human resource constraints and lack of professional preceptors [36]. Congestion of students seeking practice opportunities in a single health facility makes it difficult to expose them to adequate case-load [37]. It is important to clarify that midwifery schools are accountable for ensuring clinical practice opportunities of adequate quality and competency building of their students [38]. The standards of 40 births attended by students was not consistently met; for comparison a third of midwifery students in Ethiopia met their standard of only 20 births [24]. Caseloads in many hospitals are high and it is achievable in Afghanistan to ensure students attend 40 births. It needs, however, commitment to students working 24/7 and improved coordination of student placements. These issues can be addressed by ANMC through revised accreditation processes and addressing socio-cultural barriers.

Inconsistency of curricula in different schools is a chronic issue with only five among the nine IHS schools using the latest curriculum in 2011 [39]. The now obsolete 18-months CME 2010 curriculum inadvertently resulted in the misconception that CME graduates are less qualified than IHS ones. Schools should implement the latest national standard curriculum, and ANMC and MoPH should establish verifiable routines and information management systems to monitor and mitigate any deviations [5]. In Afghanistan, where SBAs include midwives, obstetricians and female general practitioners trained in Emergency Obstetric and Newborn Care (EmONC), midwives are more evenly distributed geographically among all SBAs, and provide 42% of all maternal and newborn healthcare [10, 12]. Competency-based midwifery education with adequate clinical practice is required for producing a competent workforce [40]. Midwives want better education, including access to higher education and development, to be empowered to support quality, equity and dignity as healthcare priorities [41].

### Limitations

This study was a rapid assessment conducted in purposively selected midwifery schools. Only a fraction of all private schools could, however, participate. Very few students who reported lack of access to some facilities, dared to mention access barriers indicating biased responses in favor of the schools. Very few schools were willing to share logbooks of their students. Therefore, caution is advised in generalizing the findings, especially those of private midwifery schools. The study was implemented at the end of 2017 and the findings were presented in several occasions to MoPH, AMA and other part ners in 2018 and were actively used for improvement of midwifery education. Specifically, the findings were used to expedite establishment of ANMC. However, the authors believe that the Afghan experience presented in this manuscript will still provide valuable insights. They will also provide a point of departure for any future study into the state of midwifery in Afghanistan.

## Conclusion

Strong competent midwives have the potential to transform and improve the quality of maternity care for strengthening reproductive, maternal and neonatal health in Afghanistan as well as to contribute to building a resilient health system. MoPH and ANMC need to prioritize and prepare an action plan to strengthen high-quality midwifery education and make strategic decisions on midwifery education, its management and compliance with educational standards through accreditation and enabling educational environments.

## Supplementary Information

**Additional file 1.**

## Data Availability

The corresponding author is willing to provide the data on request.

## Abbreviations

AHS: Afghanistan Health Survey
AMA: Afghan Midwives Association
AMNEAB: Afghanistan Midwifery and Nursing Education Accreditation Board
ANMC: Afghanistan Nurses and Midwives Council
BPHS: Basic Package of Health Services
CME: Community Midwifery Education
EmONC: Emergency Obstetric and Newborn Care
EPHS: Essential Package of Hospital Services
GDHR: General Directorate of Human Resources
ICM: International Confederation of Midwives
IHS: Institute of Health Sciences
KMU: Kabul Medical University
MMR: Maternal mortality ratio
MoPH: Afghanistan Ministry of Public Health
NSIA: National Statistics and Information Authority.
SBA: Skilled Birth Attendants
WHO: World Health Organization
