# Supplementary material for: Quality of pre-service midwifery education in public and private midwifery schools in Afghanistan: a cross sectional survey
Source: BMC Med Educ. 2022 Jan 16;22:39. doi: 10.1186/s12909-021-03056-1 (PMC8761336; doi:10.1186/s12909-021-03056-1)
Supplement: Supplementary file 1 — Additional file 1. [file 12909_2021_3056_MOESM1_ESM.docx]

**Supplementary file**

Name of the recommended textbooks for midwifery schools:

1) Kinzie, B., Gomez, P., 2004. Basic maternal and newborn care: a guide for skilled providers. Jhpiego, Baltimore, Maryland, USA

2) World Health Organization (WHO), 2000. Managing complications in pregnancy and childbirth: a guide for midwives and doctors. WHO, Geneva

3) WHO, 2003. Managing newborn problems: a guide for doctors, nurses, and midwives, WHO, Geneva

4) Klein, S., Miller, S., Thomson, F., 2004. A book for midwives: care for pregnancy, birth, and women’s health. Hesperian Foundation, Berkeley, California, USA

5) Jhpiego, 2002. Infection prevention guidelines. Jhpiego, Baltimore, Maryland, USA

6) Jhpiego, 2000. Guidelines for performing breast and pelvic examinations. Jhpiego, Baltimore, Maryland, USA

7) WHO, 2007. Family planning: a global handbook for providers, WHO, Geneva

8) Bennett, V.R., Brown, L.K., 1999. Myles textbook for midwives. 13th ed. Churchill Livingstone, Edinburgh

9) Kavle, J., 2006. Nutrition of Afghan women and children.
